# MEASURING ANKLE FLEXIBILITY IN OLDER HOME CARE RECIPIENTS WITH DISABILITIES IN THEIR HOME SETTING

**DOI:** 10.1093/geroni/igad104.2459

**Published:** 2023-12-21

**Authors:** Jordan Skowronski

**Affiliations:** University of Illinois Chicago, Chicago, Illinois, United States

## Abstract

Ankle flexibility is critical for daily activities, such as climbing stairs. Ankle range of motion (AROM), or the range through which the ankle joint can bend up and down, decreases with age. Thus, interventions to improve or maintain AROM is an important goal in geriatric rehabilitation. Evaluating the outcome of such interventions requires AROM measures. However, existing measures either require large equipment or for the participant to stand and lunge, and so are not appropriate nor practical for home-bound older adults. This paper aims to (1) describe how we developed an AROM measure for frail, older adults as part of the Promoting Seniors’ Health with Home Care Aides (Pro-Home) Study, and (2) examine the convergent and discriminant validity of the AROM measure. Data came from the baseline assessment of older home care participants’ physical function (N=115). AROM was moderately correlated with lower extremity function as measured by the Short Physical Performance Battery, r(113) = 0.30, p< 0.01 and the gait speed component scores, r(113) = 0.33, p< 0.01, and AROM was not correlated with self-reported upper extremity function. Results from the robust regression analyses are that 1 standard error unit greater averaged AROM is associated with 0.06 points higher SPPB score (p< 0.00) and 0.06 greater Gait Speed Score (p< 0.00), after adjusting for age, gender, and ambulatory device use. The significant associations between AROM and SPPB and AROM and gait speed indicate that the AROM measure has convergent validity, while the nonsignificant associations among AROM and upper extremity function show its discriminant validity.

